# Systematic Analysis of Transcriptomic Profile of the Effects of Low Dose Atropine Treatment on Scleral Fibroblasts using Next-Generation Sequencing and Bioinformatics

**DOI:** 10.7150/ijms.38571

**Published:** 2019-11-09

**Authors:** Yu-Ting Hsiao, Wei-An Chang, Ming-Tse Kuo, Jung Lo, Hsien-Chung Lin, Meng-Chi Yen, Shu-Fang Jian, Yi-Jen Chen, Po-Lin Kuo

**Affiliations:** 1 Graduate Institute of Clinical Medicine, College of Medicine, Kaohsiung Medical University, Kaohsiung 807, Taiwan; 2 Department of Ophthalmology, Kaohsiung Chang Gung Memorial Hospital, Kaohsiung 833, Taiwan; 3 School of Medicine, College of Medicine, Kaohsiung Medical University, Kaohsiung 807, Taiwan; 4 Division of Pulmonary and Critical Care Medicine, Kaohsiung Medical University, Kaohsiung 807, Taiwan; 5 Department of Ophthalmology, Kaohsiung Chang Gung Memorial Hospital and Chang Gung University College of Medicine, Kaohsiung 833, Taiwan; 6 Department of Ophthalmology, Kaohsiung Medical University Hospital, Kaohsiung 807, Taiwan; 7 Department of Emergency Medicine, Kaohsiung Medical University Hospital, Kaohsiung Medical University, Kaohsiung 807, Taiwan; 8 Department of Physical Medicine and Rehabilitation, Kaohsiung Medical University Hospital, Kaohsiung 807, Taiwan; 9 Center for Cancer Research, Kaohsiung Medical University Kaohsiung 807, Taiwan

**Keywords:** atropine, sclera, fibroblast, myopia, next-generation sequencing, bioinformatics, microRNA, messenger RNA

## Abstract

This study has two novel findings: it is not only the first to deduct potential genes involved in scleral growth repression upon atropine instillation from a prevention point of view, but also the first to demonstrate that only slight changes in scleral gene expression were found after atropine treatment as side effects and safety reasons of the eye drops are of concern. The sclera determines the final ocular shape and size, constituting of scleral fibroblasts as the principal cell type and the major regulator of extracellular matrix. The aim of our study was to identify differentially expressed genes and microRNA regulations in atropine-treated scleral fibroblasts that are potentially involved in preventing the onset of excessive ocular growth using next-generation sequencing and bioinformatics approaches. Differentially expressed genes were functionally enriched in anti-remodeling effects, comprising of structural changes of extracellular matrix and metabolic pathways involving cell differentiation. Significant canonical pathways were correlated to inhibition of melatonin degradation, which was compatible with our clinical practice as atropine eye drops are instilled at night. Validation of the dysregulated genes with previous eye growth-related arrays and through microRNA-mRNA interaction predictions revealed the association of hsa-miR-2682-5p-*KCNJ5* and hsa-miR-2682-5p-*PRLR* with scleral anti-remodeling and circadian rhythmicity. Our findings present new insights into understanding the anti-myopic effects of atropine, which may assist in prevention of myopia development.

## Introduction

The sclera is a highly organized connective tissue that comprises the major component of the outermost layer of the eye. Besides providing intraocular protection and anchorage for extraocular muscles, the sclera is a crucial determinant of the final shape and size of the eye 1. The scleral structure is predominantly made up of collagen, and interwoven fibroblasts that are responsible for the regulation and production of the extracellular matrix (ECM). Enzymes that are responsible for the remodeling of the ECM, including matrix metalloproteinases (MMPs) and tissue inhibitor of metallopreoteinase (TIMP) families, and a wide variety of cytokines and signaling molecules, are important to homeostasis of the sclera 2,3.

Any change regarding eye size are facilitated by alterations in scleral tissue volume and composition. Given the important role of axial eye size in the process of emmetropization and the occurrence of myopia, the sclera has garnered much attention in ocular and refractive development research 4. Myopia is currently the most common ocular disorder, and has emerged as a major socioeconomic public health concern worldwide 5,6. Although corrective measures can be arranged by prescribing spectacle lenses, highly myopic eyes, defined as refractive error of at least -6.00 diopters (D) or axial length 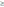
26.5mm 7, are prone to eye diseases such as myopic maculopathy, retinal detachment, cataract, glaucoma, and ultimately, blindness 8,9.

A considerable amount of effort has been devoted to understanding the biomechanical and genetic regulators in ocular growth, and preventing excessive growth of the eye 10-12. The development of myopia is accompanied by increased scleral matrix remodeling, including scleral thinning, fibril disassembly, and reduced glycosaminoglycan (GAG) and collagen content 2,13. Therefore, disrupting the scleral remodeling process may allow us to retard, or even prevent excessive ocular growth.

Anticholinergics have shown potential anti-remodeling effects of airway and lung tissues in both *in vivo* and *in vitro* studies 14,15. Antimuscarinics also demonstrated protective effects in bladder remodeling in bladder outlet obstruction situations through direct antagonistic effect and reduced muscarinic receptor expressions 16. Atropine is a non-selective antimuscarinic agent evident to be effective in preventing the progression of myopia in children 17,18, and a lower concentration of topically administered atropine could prevent myopia onset in premyopic children with lower incidence of adverse effects such as photophobia and blurry vision 19. Reports indicate that atropine could have biochemical effects on the retina or sclera, which sequentially affects sclera remodeling 1,17. However, the exact mechanism of atropine in myopia control remains unclear. Originally, inhibition of accommodation was believed to be the primary factor in preventing myopic progression 20,21. Other theories to explain more recently included potential mechanisms through neurochemical cascade initiated from muscarinic receptors, direct effect on scleral fibroblasts by inhibiting GAG synthesis 18, and chronic inflammation related to myopia development that may be downregulated by atropine 22.

Studies that target scleral interventions for preventing myopia onset are still nascent 1, and detailed mechanisms remain unclear. Previous study suggested dose-dependent cytotoxicity of atropine to human corneal epithelial cells at concentrations above 0.03% 23, but the cytotoxic effect to scleral fibroblasts is uncertain. We postulated the administration of very low dose atropine to scleral fibroblasts could minimize the risk of adverse effects, potentiating its preventive role in clinical use for myopia prevention in children. To explore the effects of atropine on gene expression modulation in scleral fibroblasts, we conducted this study with next-generation sequencing (NGS) technology and bioinformatics analyses. To our knowledge, this is the first study to systematically investigate the changes of gene regulation in scleral fibroblasts treated with atropine.

## Materials and Methods

### Study Design

**Figure 1:**
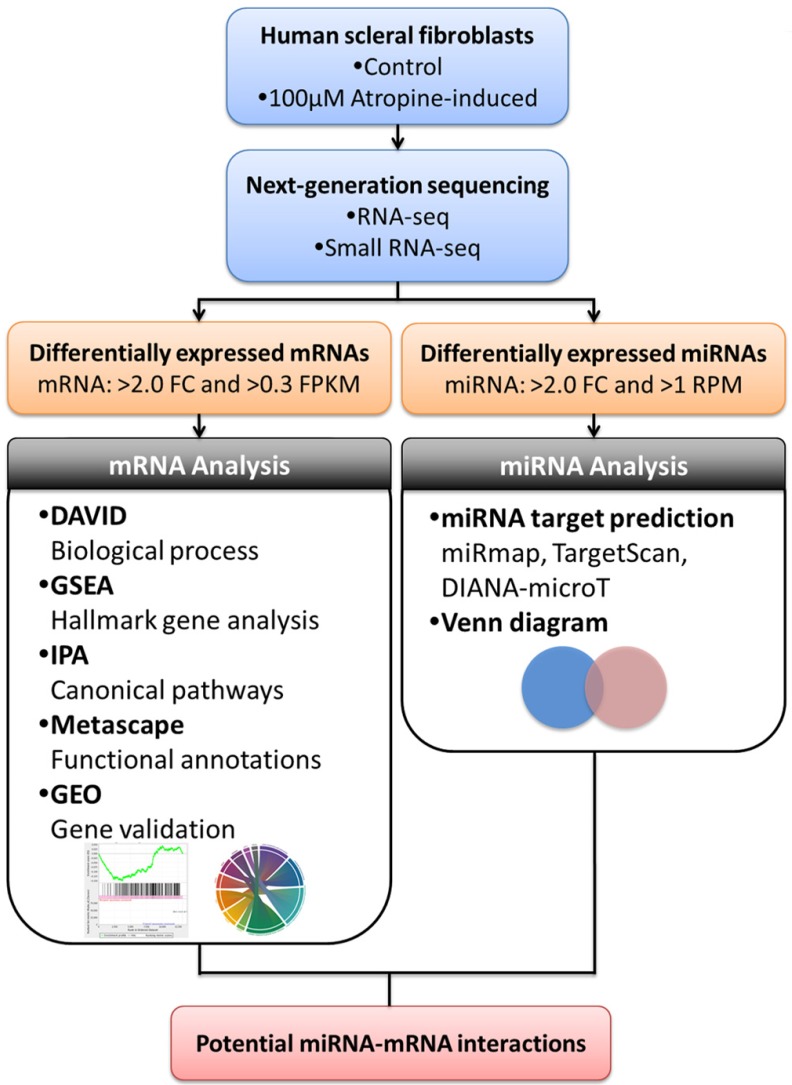
** Flowchart of study design.** Scleral fibroblasts were cultured with 0.1% DMSO (control) or 100
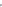
M atropine for 24 hours, and were harvested for RNA and small RNA deep sequencing. The differentially expressed genes were analyzed for enrichment analyses using various bioinformatics databases, and verified in representative arrays in GEO database. Putative targets of differentially expressed miRNAs were predicted with miRNA prediction databases, and potential miRNA-mRNA interactions were determined through Venn diagram. The potential miRNA-mRNA interactions were then validated by other miRNA prediction databases.

The study flowchart is illustrated in Figure 1. Scleral fibroblasts (the first passage) were cultured with 0.1% DMSO (control) and 100
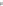
M atropine 22,24,25 for 24 hours. The fibroblasts were then harvested for RNA and small RNA sequencing through the NGS platform. The differentially expressed genes (>2.0 fold-change) were analyzed using bioinformatics tools including the Database for Annotation, Visualization and Integrated Discovery (DAVID) database 26, Gene Set Enrichment Analysis (GSEA) software 27, Ingenuity^®^ Pathway Analysis (IPA) 28, and Metascape 29 for pathway analysis and functional interpretation. Next, these differentially upregulated and downregulated genes were verified in representative Gene Expression Omnibus (GEO) datasets 30. The target prediction for the differentially expressed microRNAs (miRNAs) (>2.0 fold-change) were analyzed with miRmap 31, and genes with potential miRNA-mRNA interactions were determined through Venn diagram (http://bioinformatics.psb.ugent.be/webtools/Venn/). These potential miRNA-mRNA interactions were further confirmed by other prediction databases, TargetScan 32 and DIANA-microT 33. Lastly, an English literature search for the associated functions of these dysregulated genes was carried out to generate the hypothesis.

### Culture of Primary Cells

Human scleral fibroblasts (Part No. FC0098, Lot No. 06992) were purchased from Lifeline Cell Technology. The cells were incubated at 37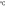 in a 5% CO_2_-containing incubator in FibroLife^®^ S2 LifeFactors kit (Lifeline Cell technology, Catalog No. LS-1038) containing 0.5 mL recombinant human FGF-basic (rh FGF-b), 0.5mL recombinant human insulin, 0.5 mL ascorbic acid, 18.75mL L-glutamine, 0.5mL hydrocortisone hemisuccinate, 10mL fetal bovine serum (FBS), 0.5mL Gentamicin and Amphotericin B (G-A). The medium was changed every 2 to 3 days and the cells were harvested at 80-90% confluence.

### Atropine Treatment

Atropine powder with a purity 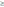
99%, purchased from Sigma-Aldrich (St. Louis, MO, USA), was dissolved in dimethyl sulfoxide (DMSO). In atropine-treated condition, scleral fibroblasts were treated with 100
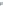
M atropine for 24 h. In control condition, scleral fibroblasts were treated with 0.1% DMSO, the carrier solvent. The atropine concentration at 100
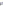
M was based on previous studies that evaluated the effects of atropine on scleral fibroblasts and ocular cell types 22,24,25. To avoid losing original characteristics of scleral fibroblasts with serial passages, the first passages of cells following cultivation from primary cells underwent treatment and were harvested for NGS analysis.

### Next-Generation Sequencing (NGS) for miRNA and mRNA Expression Profiling

The NGS technique was used for examining the expression profiles of miRNAs and mRNAs as described in our previous studies 34-36. Total RNA from the atropine-treated and normal scleral fibroblasts were extracted with TRIzol^®^ Reagent (Invitrogen, USA.) at Welgene Biotech Co., Ltd. (Taipei, Taiwan). Purified RNA was quantified at an optical density of 260nm with a ND-1000 spectrophotometer (Nanodrop Technologies, Wilmington, DE, USA). The quality of extracted RNA was analyzed using a Bioanalyzer 2100 (Agilent Technologies, Santa Clara, CA, USA), with RNA 6000 LabChip^®^ kit (Agilent Technologies, Santa Clara, CA, USA). The small RNA library construction and NGS were executed by Welgene Biotech Co., Ltd. (Taipei, Taiwan). Samples were prepared with the QIAseq miRNA Library Kit (QIAGEN) following the instruction manual. Following the sequential ligation of adaptors to the 3' and 5' ends of miRNAs, universal cDNA synthesis was carried out, and the cDNA constructs containing 18-40 nucleotide (140-155 nucleotides in length with both adaptors) RNA fragments were chosen. The libraries were sequenced on an Illumina platform (75-cycle single-end read). Sequencing data was processed by Illumina software. The small RNA sequencing data was further analyzed through a filtering process to procure qualified reads. Trimmomatic (version 0.36) 37 was applied to trim and discard reads with low quality scores. By using miRDeep2 38, qualified reads were examined, and then the reads were aligned to the reference genome downloaded from UCSC (University of California, Santa Cruz, CA, USA) 39. Since miRNAs usually map to few genomic locations, only reads that mapped perfectly to the genome five or less times were used for miRNA detection. The miRNAs with low levels (<1 normalized read per million (rpm)) were excluded, and those with >2-fold changes were considered dysregulated.

For transcriptome sequencing, library construction with Agilent's SureSelect Strand Specific RNA Library Preparation kit (Agilent Technologies, Inc.) followed by sequencing the library on a Solexa platform (150 paired-end cycles) using the TruSeq sequencing-by-synthesis (SBS) kit (Illumina, Inc., San Diego, CA, USA) was carried out. Trimmomatic (version 0.36) 37 was also implemented to trim and eliminate reads with low quality score. Qualified reads were then surveyed using HISAT2 40. The genes with low expression levels (<0.3 fragment per kilobase of transcript per million mapped reads (FPKM)) in both atropine-treated scleral fibroblasts and normal scleral fibroblasts were excluded, and those with >2 fold-changes were considered as differentially expressed genes.

### miRmap Database Analysis

miRmap is an open-source software library for predicting miRNA targets 31. This miRNA target prediction tool uses thermodynamic, conservation, probabilistic and sequence-based approaches for the prediction of repression strength. With the predictive reference value, the miRmap score, a list of putative target genes could be identified. A higher miRmap score indicates higher repression strength. In this study, miRmap score 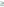
97.0 was used as the criteria for putative miRNA target selection.

### DIANA-microT Analysis

DIANA-microT v5.0 is an online database that is customized for miRNA target prediction and functional analysis 33. This new version of the microT server uses an enhanced target prediction algorithm, DIANA-microT-CDS, and has incorporated miRBase version 18 and Ensembl version 69. microT-CDS is the only algorithm online that is peculiarly designed to identify targets on a positive and a negative set of miRNA Recognition Elements (MREs), located in both the 3'-UTR and CDS regions.

### TargetScan Database Analysis

TargetScan is web application that predicts the target of miRNA by searching for the presence of conserved 8, 7, and 6 mer sites matching the seed region of each miRNA 32. Ranking of the prediction results are established on the predicted efficacy of targeting or the probability of conserved targeting.

### DAVID Database Analysis

The Database for Annotation, Visualization and Integrated Discovery (DAVID) is a powerful gene functional annotation tool 26. It merges Gene Ontology (GO) and Kyoto Encyclopedia of Genes and Genomes (KEGG) pathway, and a list of genes of interest can be categorized into clusters of related biological process, cellular components, and molecular functions by calculating the similarity of global annotation profiles with an agglomeration algorithm method. DAVID database provides an Expression Analysis Systematic Explorer (EASE) score, a modified Fisher's Exact *p* value for reference. The EASE score stands for how particularly the user genes are involved in the category, and we selected EASE score=0.1 as default in this study.

### GSEA Database Analysis

The Gene Set Enrichment Analysis (GSEA) software interprets and analyzes gene expression data based on gene sets, in other words, groups of genes that have common biological function, chromosomal location, or regulation 27. GSEA considers all genes in an experiment, not only appointed genes with significant differential expression. Furthermore, GSEA provides a more accurate null model by assessing the significance by permuting phenotypes, which preserves gene-gene correlations. In the present study, GSEA desktop version 3.0 was used for analysis.

### Ingenuity Pathway Analysis (IPA)

The Ingenuity Pathway Analysis (IPA) (Qiagen Inc., Valencia, CA, USA) is a web-based bioinformatics application that provides a comprehensive interpretation of functional analysis, integration, and visualization from high-throughput experiments including RNA sequencing, small RNA sequencing, microarrays, metabolomics and proteomics 28. Identification of predicted canonical pathways, associated diseases and function, key upstream regulators, and related signaling pathways can be obtained after the gene list of interest is uploaded to IPA for core analysis. IPA can also construct casual networks to generate mechanistic hypotheses, established from changes of expression in the dataset. The dysregulated genes from the scleral fibroblasts were uploaded to IPA (version 2.3) to identify the associated canonical pathways.

### Metascape Analysis

Metascape is a web-based portal that engineers a knowledgebase synchronization pipeline to analyze and interpret large-scale datasets 29. Metascape facilitates gene annotation integration, functional enrichment, interactome analysis, membership analysis, and multi-gene-list meta-analysis. It provides a convenient one-click Express Analysis interface to generate interpretable results. Metascape version 3.5 was used for analyzing the candidate genes in scleral fibroblasts for functional profiles. Graphical representations of functional relationships were visualized using Flourish (https://flourish.studio).

### GEO Database Analysis

The GEO database provides public access to high-throughput gene expression data of microarrays, chips or NGS 30. The database can be linked to GEO2R, a web-based tool, where users can carry out further analysis by acquiring the expression values of genes of interest. The arrays related to eye growth in human ocular tissues (GSE71743 and GSE18811) were used in this study to identify genes that expressed in opposing directions with our NGS results.

## Results

### Gene Expression Profiles and miRNA Changes in Scleral Fibroblasts

The mRNA and small RNA expression data of atropine-treated scleral fibroblasts and normal scleral fibroblasts were sequenced using an NGS platform. Gene expression analysis revealed 389 differentially expressed genes with at least a 2.0-fold change, inclusive of 215 upregulated and 174 downregulated genes. miRNA expression analysis yielded a total of 23 miRNAs with fold-change >2, which included 15 upregulated and 8 downregulated miRNAs.

### Gene Ontology Analysis of Dysregulated genes in Scleral Fibroblasts Treated with Atropine

**Figure 2:**
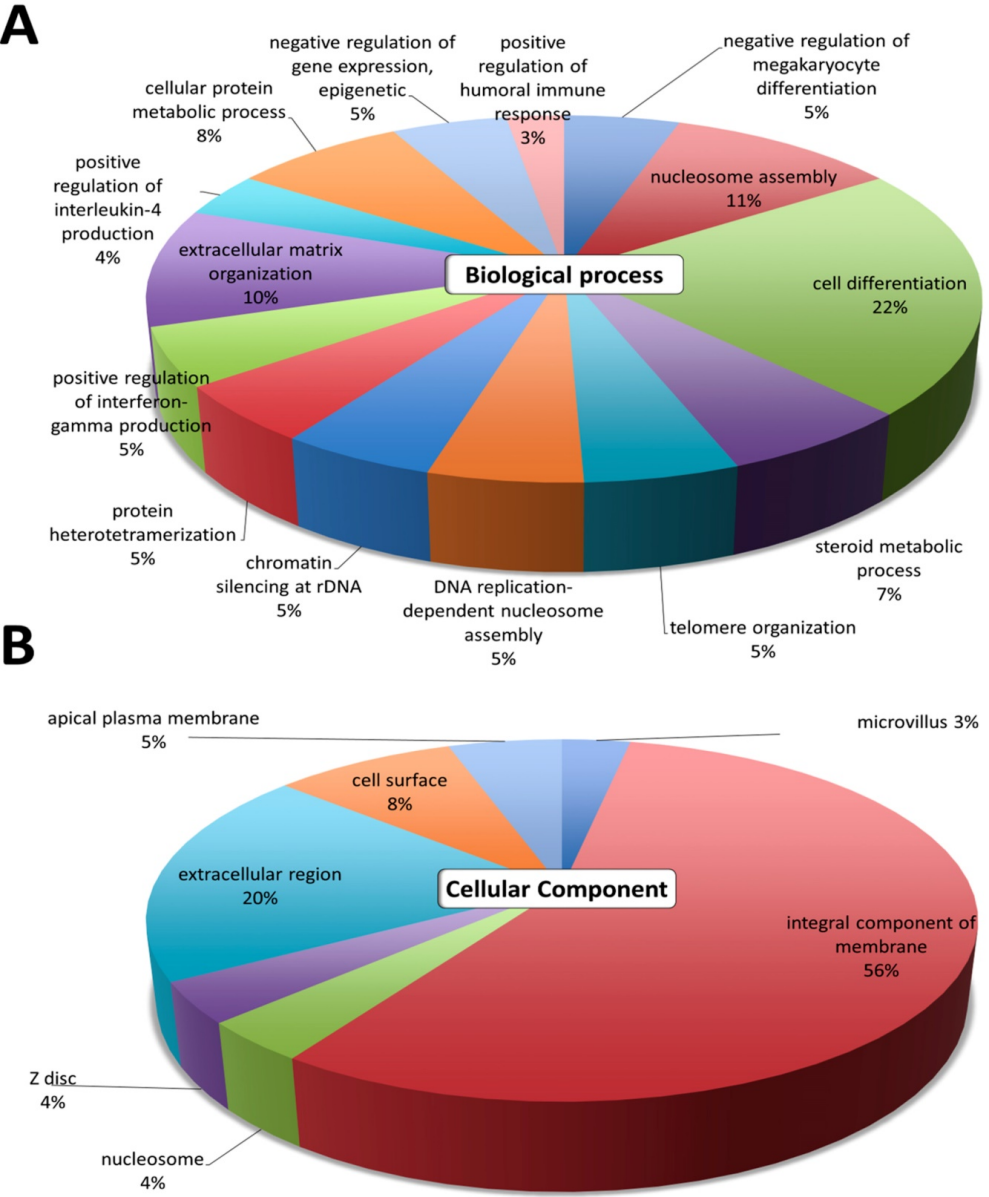
** Differential expression analysis of genes in atropine-treated scleral fibroblasts.** Gene ontology terms involved in the 389 differentially expressed genes of atropine treated scleral fibroblasts. The 389 genes were input into the DAVID database to determine related **(A)** biological functions and **(B)** cellular component domain. The selected criteria for functional annotation analysis were EASE score=0.1 and *p* value < 0.05. The area of each pathway reflected the number of genes involved.

The gene ontology analyses of the 389 differentially expressed genes were first analyzed using DAVID. The top GO terms in the 'Biological Process' were cell differentiation, nucleosome assembly, extracellular matrix organization, cellular protein metabolic process, and steroid metabolic process (Figure 2A). The results in 'Cellular Component' indicated that the most significant function was involved in the integral component of membrane and the extracellular region (Figure 2B). These GO annotations indicated the involvement of dysregulated genes in metabolic pathways involving cell differentiation, and structural changes related to the extracellular matrix.

**Figure 3:**
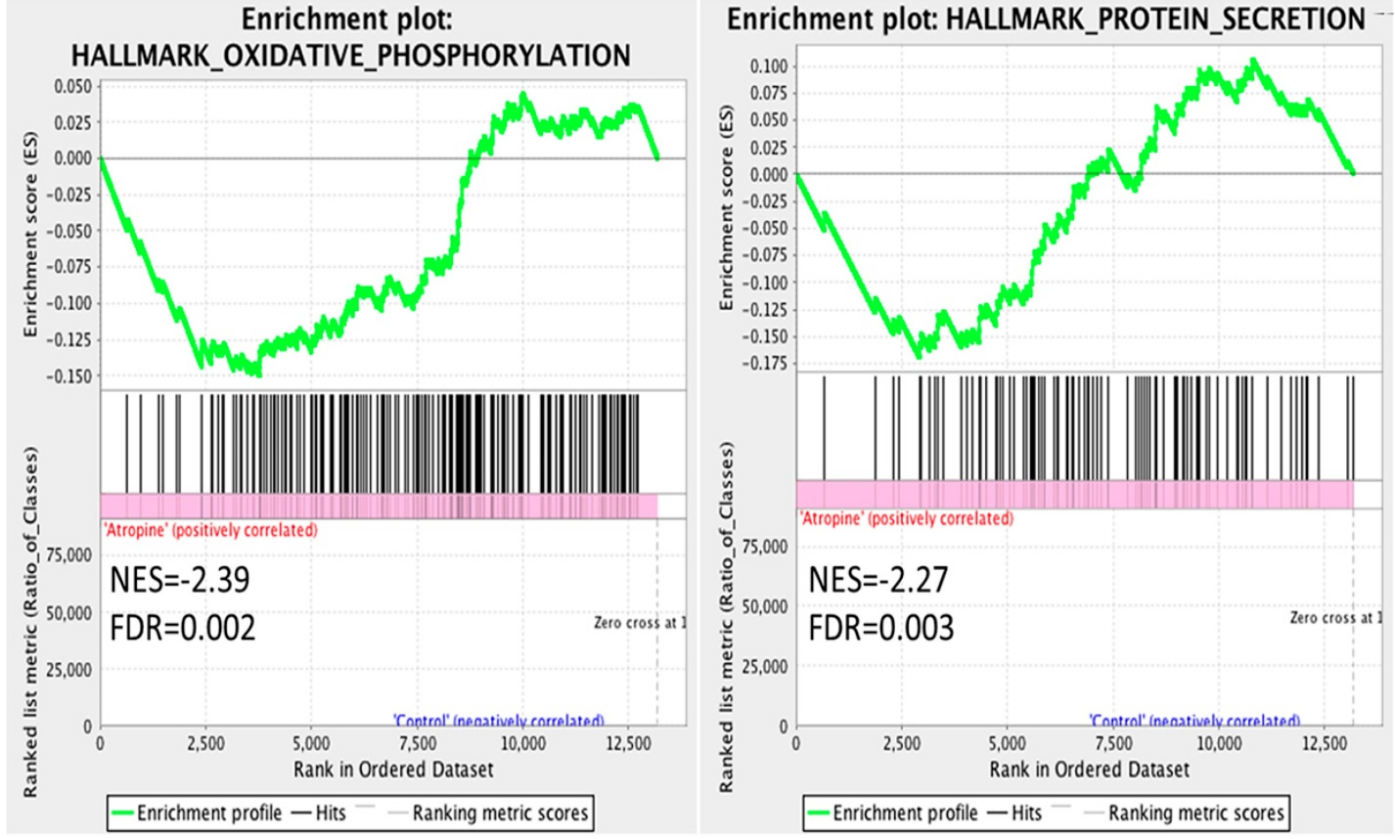
** Gene Set Enrichment Analysis (GSEA) of gene expression in scleral fibroblasts.** All the expressed genes that were >0.3 FPKM in either normal or atropine-treated scleral fibroblasts were uploaded into GSEA for enrichment analysis. The h.all.v6.2.symbols.gmt [Hallmarks] gene set database was used as the gene set collection for analysis. GSEA performed 1000 permutations and gene_set permutation was used. The maximum and minimum size for gene sets were set at 500 and 15, respectively. Cutoff for significant genes sets was false discovery rate (FDR) <5%.

The dysregulated genes were systematically assessed for functional enrichment using the GSEA database. The expression values of the genes in atropine-treated scleral fibroblasts and normal scleral fibroblasts were all uploaded into GSEA software, and analyzed with the hallmark gene sets database. A more stringent false discovery rate (FDR) cutoff was used, with the cutoff for significant genes sets as FDR <5%. The gene sets enriched in normal scleral fibroblasts included oxidative phosphorylation and protein secretion (Figure 3). Notably, the results revealed that the functions of normal scleral fibroblasts were significantly enriched in both the metabolic and structural pathways.

**Figure 4:**
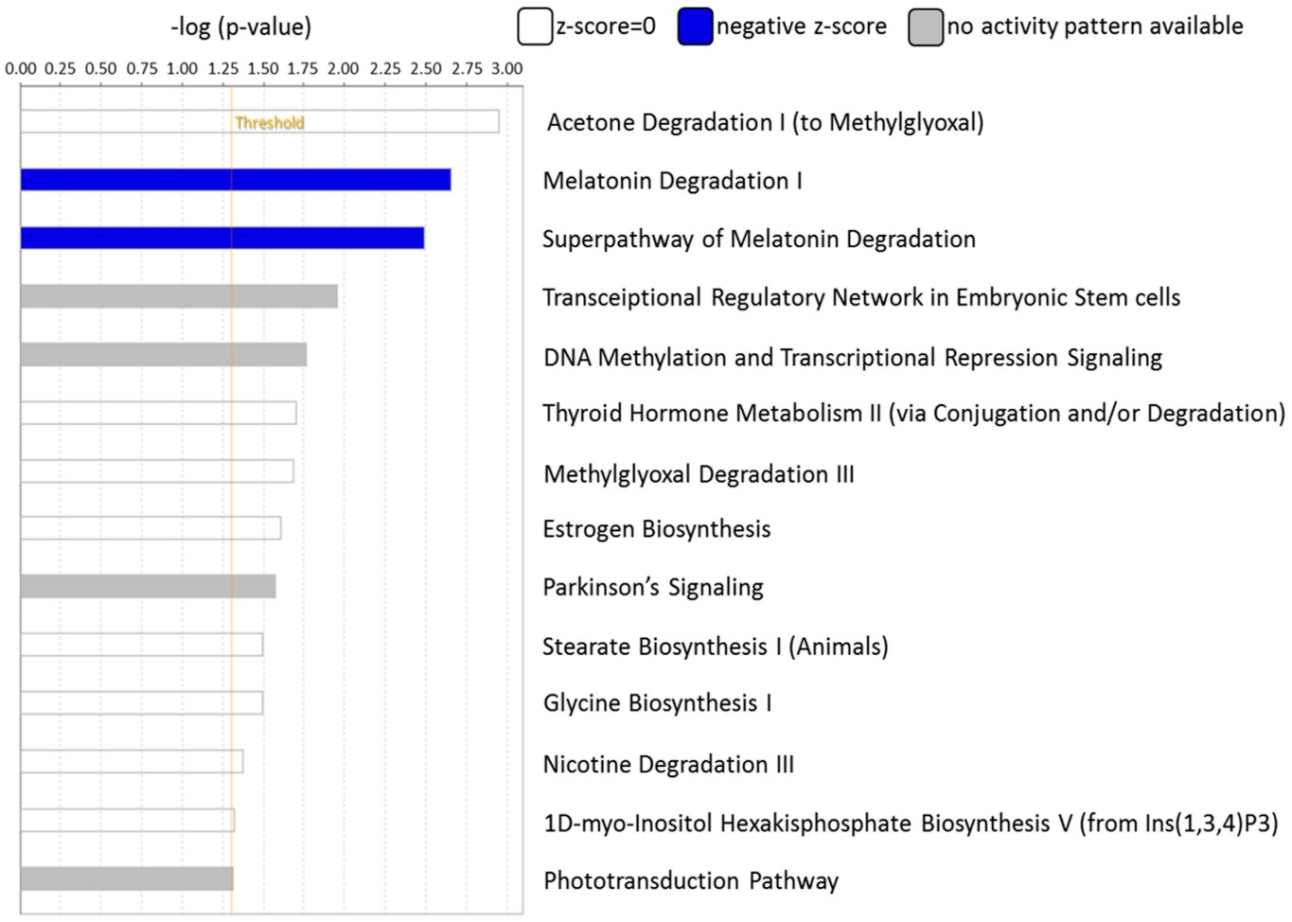
** Functional analysis of differentially expressed genes identified in atropine-induced scleral fibroblasts by Ingenuity Pathway Analysis (IPA).** Of the 14 enriched pathways, only two pathways showed a significant predicted pattern of activity. The two pathways, melatonin degradation and superpathway of melatonin degradation, were significantly inhibited.

**Figure 5:**
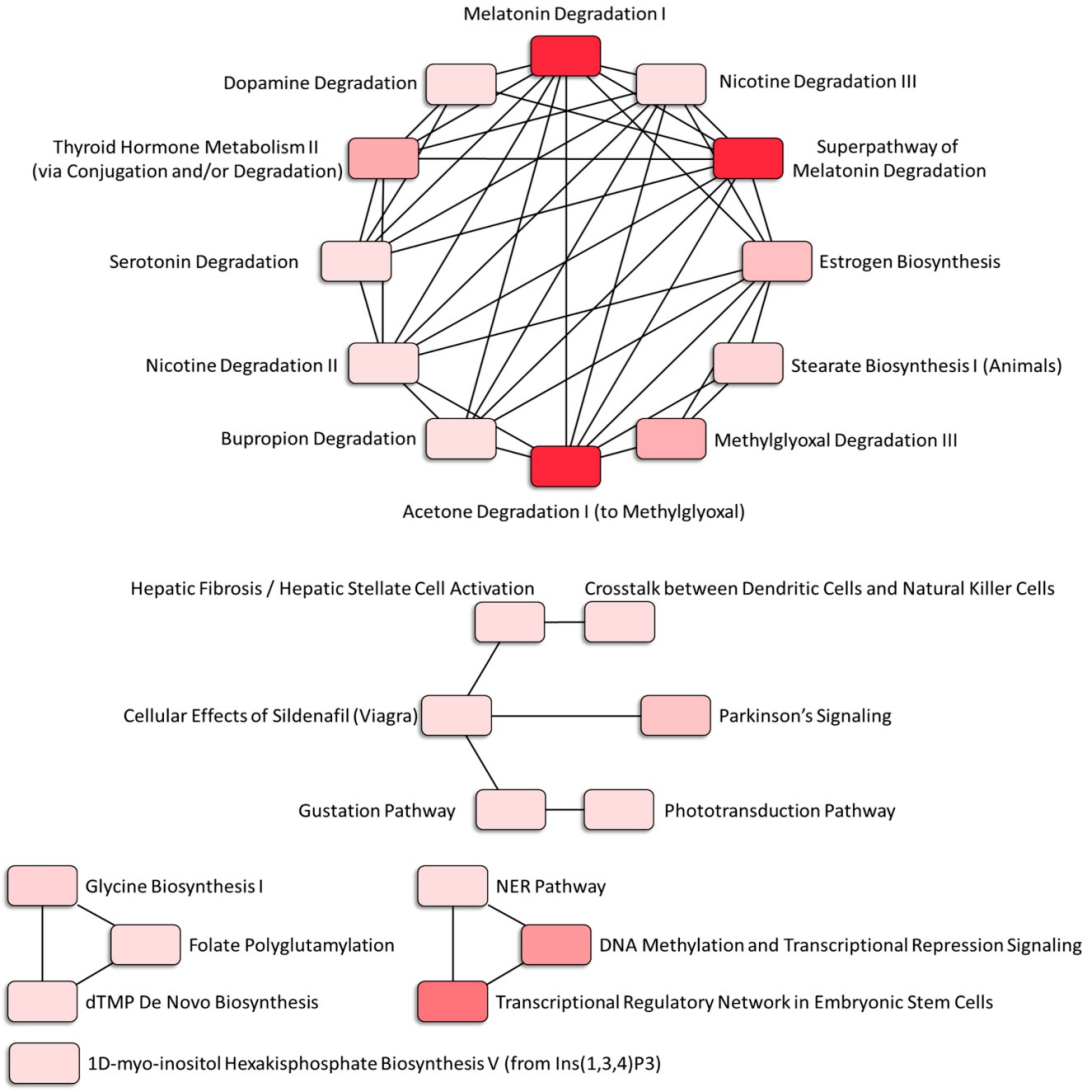
** Associated networks of pathways related to dysregulated genes in atropine-treated scleral fibroblasts.** The networks of pathways that were in relation were analyzed by Ingenuity Pathway Analysis (IPA).

The canonical pathways associated with the 389 differentially expressed genes were explored using IPA. Pathway analysis showed that the dysregulated genes were enriched in 14 pathways, with two pathways showing a significant negative prediction pattern (Figure 4). Both pathways, melatonin degradation and superpathway of melatonin degradation, were significantly inhibited. Figure 5 showed the networks of the pathways that were in association. The metabolism of dopamine and hormones were also found to be involved in the dysregulated genes, and connected with melatonin degradation pathways. This implies that the gene expression and cell homeostasis changes in atropine-treated scleral fibroblasts may be potentially regulated by melatonin-related signaling pathways.

### Identification and Functional Annotation Classification of Potential miRNA-mRNA Interactions in Atropine-Treated Scleral Fibroblasts

**Figure 6:**
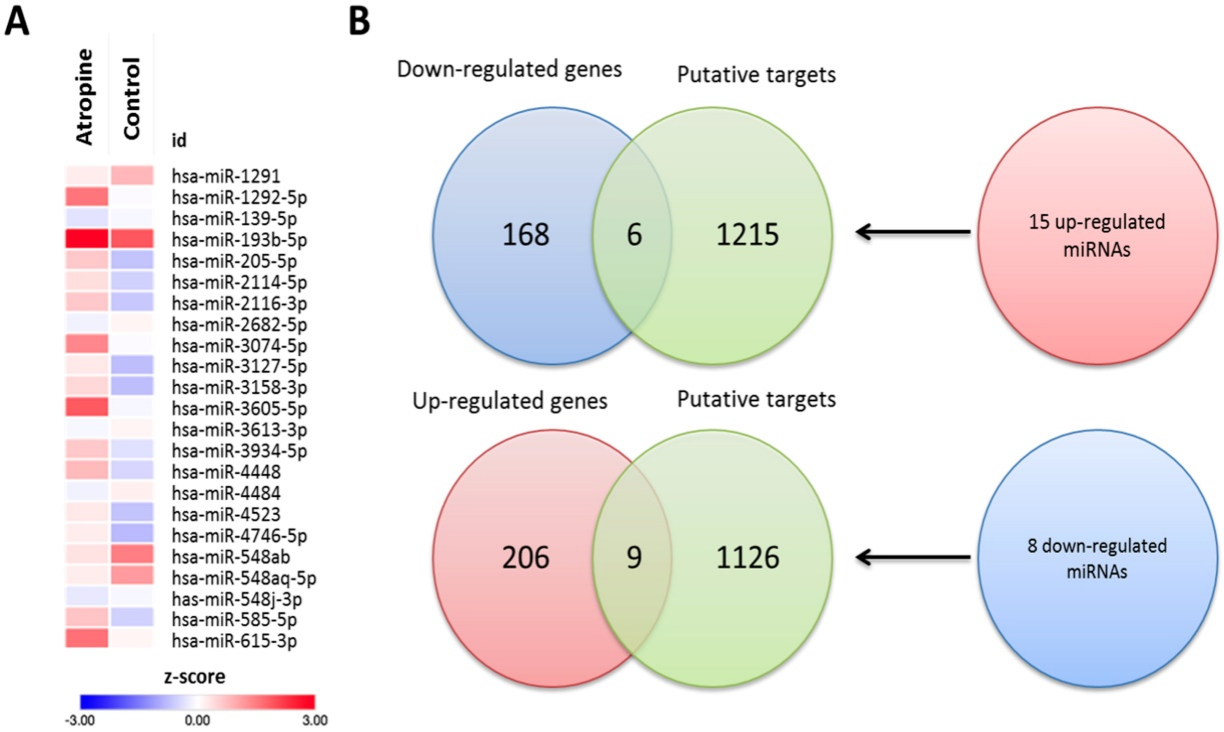
** Identification of differentially expressed miRNAs and potential miRNA-mRNA interactions in human primary scleral fibroblasts. (A)** The heatmap analysis of differentially expressed miRNAs from atropine-treated and normal scleral fibroblasts with z-score values were shown. **(B)** The 15 up-regulated and 8 down-regulated miRNAs predicted 1221 and 1135 putative targets, respectively. Putative targets of differentially expressed miRNAs were predicted using miRmap database, setting the repression score at ≥ 97.0. The candidate genes were those overlapping with differentially expressed mRNAs in atropine-treated and control scleral fibroblasts. Fifteen genes (9 upregulated and 6 downregulated) with potential miRNA-mRNA interactions were identified.

To determine the potential miRNA-mRNA interactions between normal and atropine-treated scleral fibroblasts, we first identified 23 differentially expressed miRNAs with >2.0-fold change, including 15 upregulated and 8 downregulated miRNAs. The heatmap of the dysregulated miRNAs is shown in Figure 6A. Subsequently, the putative targets of the 23 miRNAs were predicted using miRmap database, setting a selection criteria of miRmap score more than 97.0. The analytic results revealed 1221 targets of 15 upregulated miRNAs and 1135 targets of 8 downregulated miRNAs. Then, we matched these putative targets to the 389 dysregulated protein-coding genes. Using an online Venn diagram analysis, 6 downregulated and 9 upregulated genes were discovered in the intersection (Figure 6B). The 15 candidate genes with 16 potential miRNA-mRNA interactions that were identified in atropine-treated scleral fibroblasts are shown in Table 1.

**Figure 7:**
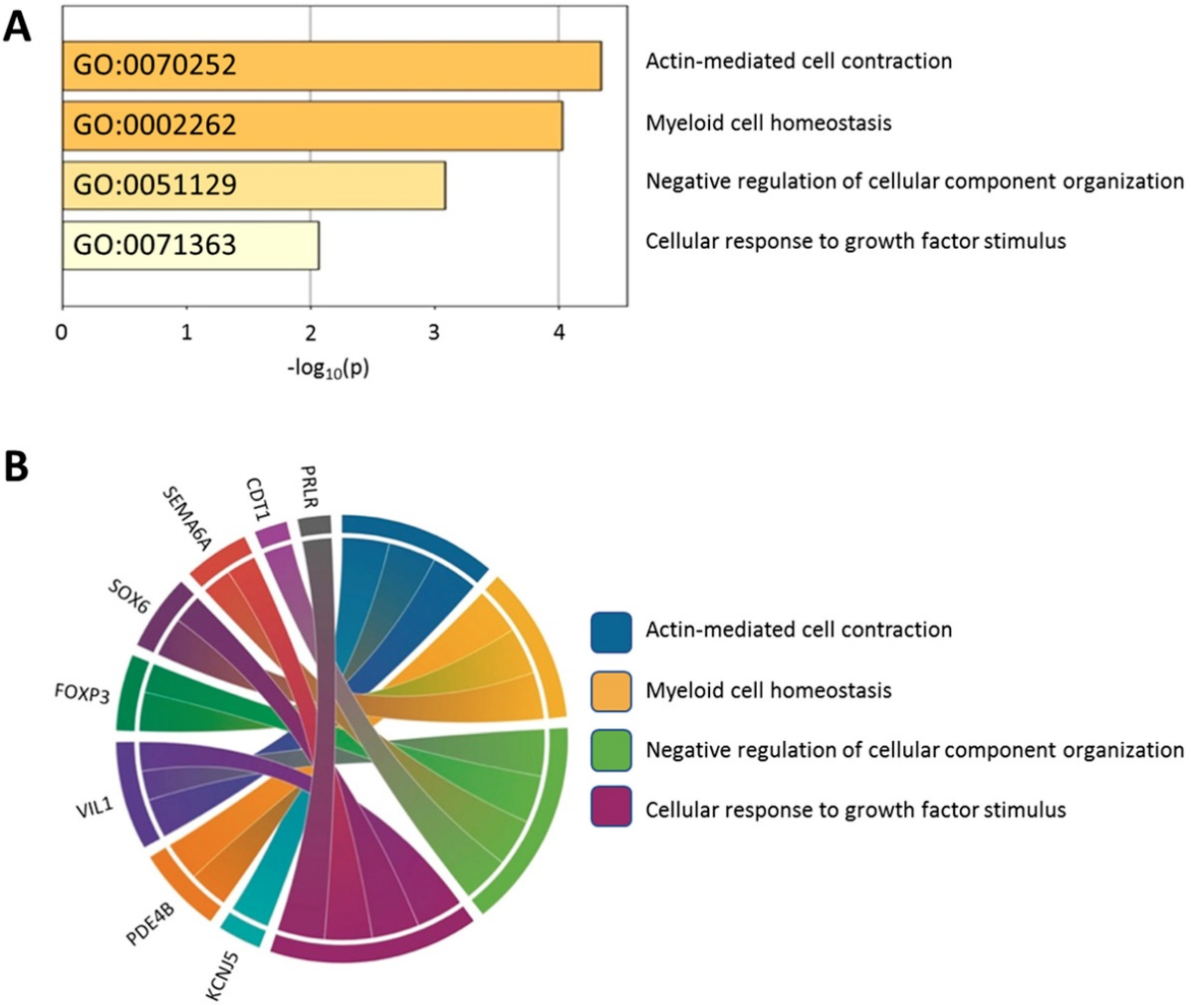
** Functionally enriched terms across candidate genes. (A)** The 15 candidate genes identified in atropine-treated and control scleral fibroblasts were analyzed by Metascape. The process enrichment analysis had been carried out with the GO Biological Processes ontology source, and all genes in the genome were used as the enrichment background. Terms with a p-value < 0.01, a minimum count of 3, and an enrichment factor > 1.5 were collected and grouped into clusters based on their membership similarities. The p-values were calculated based on the accumulative hypergeometric distribution. **(B)** Chord diagram showing the top 4 clusters of the 15 candidate genes with their representative enriched terms.

Using the Metascape database, the functional profiles of the 15 candidate genes between normal and atropine-treated scleral fibroblasts were analyzed. As shown in Figure 7A, the top four clusters were mostly involved in changes in cellular development, and regulation of structural organization. Functional annotation of the 15 candidate genes with their over-represented enriched terms are visualized graphically in Figure 7B.

### Analysis of Candidate Genes Expression Pattern in Related Ocular Tissue Arrays from Gene Expression Omnibus (GEO) Database and Identification of Potential Molecular Signatures in Ocular Growth Microenvironment

**Figure 8:**
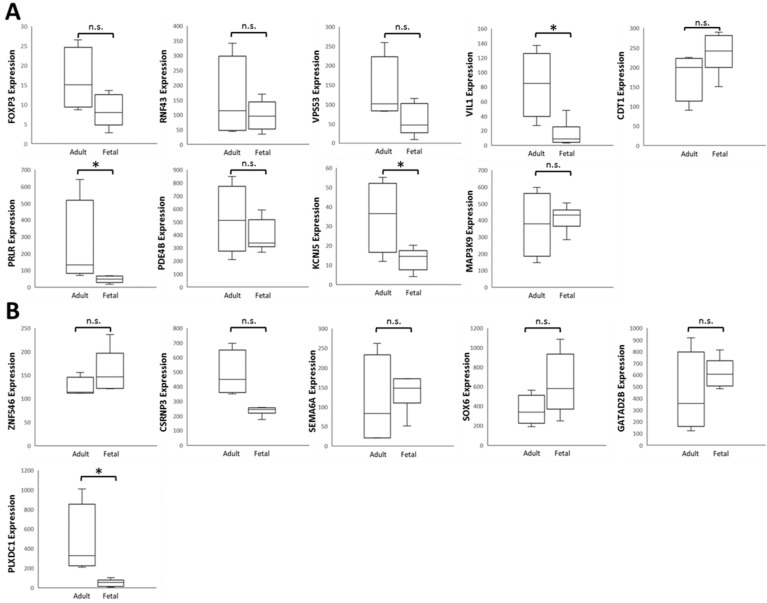
** Analysis of 15 candidate genes with potential miRNA-mRNA interactions in ocular growth-related dataset.** Expression values of **(A)** nine upregulated genes and **(B)** six downregulated genes were validated in an ocular growth-related dataset comparing fetal and adult retinal pigmented epithelial tissues derived from GEO database (GSE 18811). The significantly upregulated expressions of *VIL1, PRLR* and *KCNJ5* were in opposing directions with our atropine-induced scleral fibroblasts NGS result. * indicated p < 0.05, and n.s. indicated no statistical significance. (Probe ID reference: *FOXP3*, 221333_at; *RNF43*, 218704_at; *VPS53*, 221707_s_at; *VIL1*, 205506_at; *CDT1*, 228868_x_at; *PRLR*, 227629_at; *PDE4B*, 211302_s_at; *KCNJ5*, 208397_x_at; *MAP3K9*, 213927_at; *ZNF546*, 240429_at; *CSRNP3*, 235018_at; *SEMA6A*, 225660_at; *SOX6*, 235526_at; *GATAD2B*, 238076_at; *PLXDC1*, 214081_at).

To verify the 15 candidate genes in clinical samples from patients with ocular growth, the GEO database was explored for associated datasets. Since ocular growth is considered an event that involves various ocular tissues, especially in the posterior pole 41, we took datasets of the retina, choroid, retinal pigmented epithelium, and sclera into consideration. Under the criterion of *Homo sapiens* organism, there were two representative arrays comparing ocular tissues with eye growth (GSE71743 and GSE18811). One array compared scleral fibroblasts from the anterior and posterior pole of the sclera (GSE71743), and the other array compared retinal pigmented epithelium isolated from fetuses and adults (GSE18811). The expression patterns of the 15 candidate genes in the GSE18811 dataset are demonstrated in Figure 8.

Dysregulated genes were considered verified if they exhibited opposing directions in gene expressions related to ocular growth in both GSE71743 and GSE18811 datasets, and the expression changes were significant in at least one dataset. The results are shown in Table 2. According to the GEO analysis, we verified the upregulated prolactin receptor (*PRLR*) and potassium voltage-gated channel subfamily J member 5 (*KCNJ5*) and downregulated semaphorin 6A (*SEMA6A*) as the target genes of interest.

### Identification of Potential miRNA-mRNA Interactions of PRLR, KCNJ5, and SEMA6A in Atropine-Treated Scleral Fibroblasts

The potential miRNA regulations of *PRLR, KCNJ5*, and *SEMA6A* with miRmap score 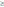
97.0 were chosen, and matched to the 23 differentially expressed miRNAs from our data. Subsequently, the 3 genes with potential miRNA-mRNA interactions were verified in two other miRNA prediction databases, TargetScan and DIANA-microT. Results yielded hsa-miR-2682-5p-*PRLR* and hsa-miR-2682-5p-*KCNJ5* interactions were validated in all three miRNA prediction databases (Table 3).

## Discussion

We report for the first time, utilizing NGS for human scleral miRNA and mRNA expression profiling upon treatment with atropine. As the sclera defines the final ocular shape and size, the sclera has become an appealing targeting tissue for myopia control 1,2. Preventing the onset of myopia and setting back its progression in early life are vital steps in management of myopia. There is no cure for myopia at present, however, nightly atropine eye drops have been prescribed for controlling progressive axial myopia since the 1960s and is still the most effective pharmacological treatment in clinical practice for myopia 17-19,42. In this study, we sought to identify differentially regulated miRNAs and gene expression in atropine-treated scleral fibroblasts and explore their potential to adjust the course of scleral remodeling as a treatment strategy to arrest ocular elongation.

**Figure 9:**
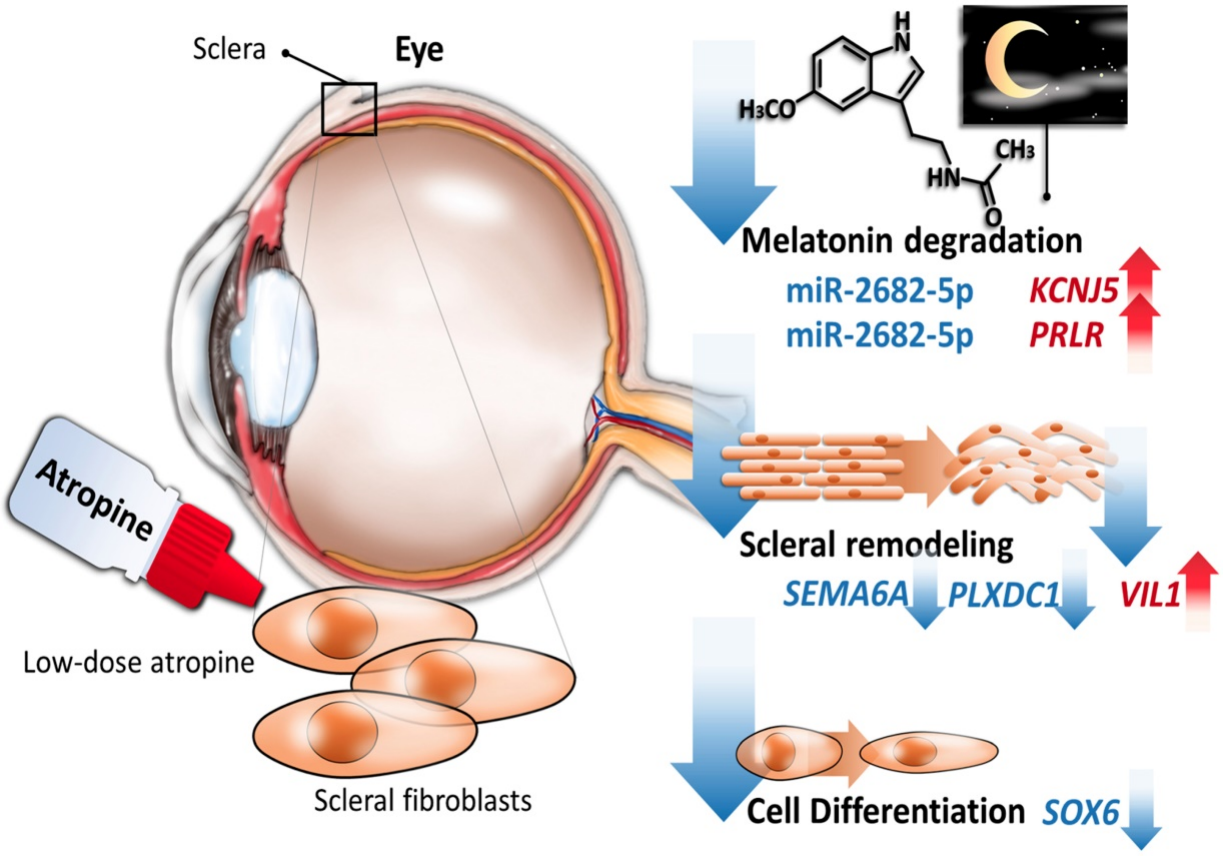
Schematic summary of the proposed molecular signatures in scleral fibroblasts after atropine treatment.

Accumulating evidence revealed that anticholinergics have anti-remodeling effects 14-16, and our results also showed that gene expression across atropine-treated scleral fibroblasts were associated with anti-remodeling effects via metabolic and structural pathways. We identified 389 differentially expressed genes in scleral fibroblasts treated with low dose atropine as compared with the control group. Gene ontology analysis revealed that genes were closely linked to metabolic regulation involving cell differentiation and oxidative phosphorylation, and structural changes comprising of ECM organization and protein secretion. In addition, canonical pathway analysis indicated that these alterations in scleral remodeling processes may be modulated by melatonin signaling pathways. Of the 23 differentially regulated miRNAs, we found two potential altered miRNA-mRNA interactions, including hsa-miR-2682-5p-*PRLR* and hsa-miR-2682-5p-*KCNJ5*, to be of importance in response to low dose atropine treatment in scleral fibroblasts. The graphic summary of these gene expression changes is presented in Figure 9.

Strong evidence demonstrated that the cycle of light and dark, and ocular circadian rhythms is essential in the maintenance of ocular axial elongation and eye growth 43-45. Melatonin, which can be secreted by pinealocytes, retinal photoreceptors, and ciliary epithelial cells, plays an important part in the coordination of the circadian system 46-48. The expression of two melatonin receptors, MT1 and MT2, have been identified in the sclera 44,49. Activation of MT1 and MT2 lead to modulation of cyclic adenosine monophosphate (cAMP) and intracellular Ca^2+^ levels 50. The circadian rhythmicity of melatonin synthesis and release was shown to control several biological rhythms in the eye, inclusive of circadian changes in intraocular pressure 51, modulation of dopamine release 52, phototransduction and photoreceptor renewal in retina 53, and as an antioxidant in lens 54. In animal models, it was speculated in the sclera of chick eyes that rhythmic fluctuations in scleral proteoglycan synthesis with a period of approximately 24 hours contributed to the rhythm in axial elongation 55. Regular intervals of light and dark periods are essential for eye growth regulation as shown in the reciprocal interaction between melatonin and dopamine. Constant light, constant dark, or a brief period of light exposure at night could result in ocular growth disruptions and subsequent refractive errors 44,56-58. In the latter, the authors suggested that stimulation in eye growth was due to acute suppression of melatonin by light at night 58. In our study, the results by z-score estimation from IPA implied the inhibited functions of melatonin degradation in atropine-treated scleral fibroblasts. Network analysis of associated canonical pathways further exhibited the correlation between melatonin regulation and dopamine metabolism. As atropine eye drops are instilled before bedtime at night, our results are concordant with findings from previous studies as inhibiting melatonin degradation in the sclera at night could potentially lead to eye growth retardation.

Our data identified potassium voltage-gated channel subfamily J member 5 (KCNJ5), a G protein-gated ion channel 59, to be a potential regulator after atropine treatment. The encoded protein had a greater tendency to allow potassium to flow into a cell rather than out of a cell, and its expression was abundant in the adrenal gland 59,60. One of the KEGG pathways of *KCNJ5* include circadian entrainment, where its orthology to Kir3, which is affected by the MT1 receptor via the G_i_ signaling-transduction proteins, was noted 61. Research has mainly focused on its role with hyperaldosteronism 62,63. Research has supported the associations between genetic variations of steroidogenesis enzyme genes and high myopia. The *KCNJ5* gene is considered a candidate SNP variation for increased risk of pathological myopia in genome-wide association analysis studies 64. Altogether, with these literature reviews, the upregulation of *KCNJ5* in our NGS results implicates a novel finding in the effects of atropine on the sclera.

Prolactin receptor (PRLR) belongs to the type I cytokine receptor family, and is a receptor for prolactin (PRL). PRLR can be activated by three human hormones, prolactin, growth hormone, and placental lactogen, therefore is responsible for a wide variety of physiological actions 65. The circadian rhythm of PRL secretion in humans had been described by Sassin and colleagues 66. PRL secretion showed a nocturnal rise and a robust sleep-independent endogenous circadian rhythm 67. *PRLR* expression in human breast cancer is correlated with good prognostic clinicopathological parameters, as expression of PRL pathway-based gene signature comprised of PRL, PRLR, Jak2 and Stat5a showed a notable association with more differentiated tumors 68. In the regulatory role of the PRLR/PRL system in chondrocyte differentiation of the human synovial fluid, activation increased the expression of extrapituitary PRL and components of ECM including type II collagen and proteoglycans 69. Together with this evidence, we propose the participation of *PRLR* in atropine-treated scleral fibroblasts, which has features associated with circadian rhythm and differentiation.

Scleral remodeling occurs as a consequence of increases in cell differentiation changes during myopia development 2,70. This view is supported by *in vivo* studies that hypoxia-inducible factor-1*α* (*HIF-1α*) was upregulated in the myopic sclera, which promoted myopia through fibroblast-to-myofibroblast transdifferentiation. It was determined that sclera hypoxic stress is a common feature in myopia 70,71. Aclidinium bromide, a long-acting muscarinic antagonist, was used in COPD patients not only because it reduces mucin hypersecretion, but also due to its inhibition of cigarette smoke-induced lung myofibroblast transdifferentiation. Our study demonstrated that the dysregulated genes were closely related to cell differentiation and functional enrichment showed a significant decrease in oxidative phosphorylation in scleral fibroblasts after atropine treatment. Additionally, chondrogenic differentiation in scleral fibroblasts and scleral stem/progenitor cells was found to play a role in myopia development 72-74. One of the candidate genes, SRY-Box 6 (*SOX6*), is a transcriptional activator that is required for the central nervous system development and chondrocyte differentiation with the involvement of bone morphogenetic protein-2 (*BMP-2*) 74. Furthermore, *SOX6* is expressed at very low levels in the adult brain, but higher in fetal brain and gliomas. It was suggested that upregulation of *SOX6* may induce transformation activity in the early stage in gliomas 75. In contrast, our findings showed that atropine downregulated *SOX6* expression in scleral fibroblasts, suggesting the reduced levels of *SOX6* expression may contribute to scleral remodeling repression. Moreover, clock gene Period-1 (*Per-1*) suppresses chondrocytic differentiation through negative regulation of the *SOX6* gene 76. These results imply the association between *SOX6* and the circadian rhythm.

The defining feature of the sclera in axial elongation is excessive degrees of thinning, involving both accelerated scleral matrix degradation and slowed production of new ECM 1,77. The orchestrators of this complex process are the activation of MMPs and decreased activity of TIMPs 3,78. Reduced production of new ECM is a result of decreased levels of collagen synthesis, particularly type I collagen, and diminished production of proteoglycans and their GAG side chains 12. At a morphological level, high myopic eyes have collagen fibers of smaller diameter, and fewer collagen fiber bundles 2,79. Atropine was shown to inhibit GAG synthesis in scleral fibroblasts via a nonmuscarinic mechanism 80. Acildinium bromide could attenuate collagen type I in CSE-induced lung fibroblasts 15. According to our NGS data, functional analysis pointed to ECM organization and decreased protein secretion as key contributors in scleral fibroblasts treated with atropine. Our current results revealed three candidate genes that were relevant to cellular structure and organization, including villin 1 (*VIL1*), semaphorin 6A (*SEMA6A*), and plexin domain containing 1 (*PLXDC1*). VIL1 is a member of a family of calcium regulated actin-binding proteins. Induction of cell stress alters the actin cytoskeleton in intestinal epithelial cells via down-regulation in the actin-binding protein VIL1 81. Our results are compatible with the changes on *VIL1* as it was up-regulated in atropine-treated cells. The *SEMA6A* gene promotes reorganization of the actin cytoskeleton and act as an axon guidance cue in the developing central nervous system 82. PLXDC1 is a protein that is highly expressed in the endothelial cells of tumors, and involved in angiogenesis and spinal cord development 83,84. An *in vivo* study indicated that highly expressed *PLXDC1* may be of consequence in the proliferation and maintenance in neovascular endothelial cells of fibrovascular membranes from patients with proliferative diabetic retinopathy 83. Our data yielded the downregulation of *SEMA6A* and *PLXDC1* gene, which may also have a hand in retarding the structural reorganization of scleral fibroblasts after atropine treatment. Although the roles of these genes in regulating the cell morphology of scleral fibroblasts remain largely unknown, these atropine-induced gene expression alterations might provide potential targets to reverse scleral remodeling and deserve further studies.

Although miR-2682-5p has no known function 85, it was predicted to target two of our candidate genes, *KCNJ5* and *PRLR*. A recent study reported miR-2682-5p was one of the most abundant miRNAs (95th percentile) in the aqueous humor of exfoliation glaucoma (XFG) patients, but not primary open-angle glaucoma (POAG) patients 86. The aqueous humor was the target of choice due to its function in maintaining intraocular pressure (IOP) levels in the anterior segment of the eye 87. In addition, XFG was considered a more severe disease than PAOG, with XFG patients exhibiting higher IOP levels and more difficult to manage clinically 88. However, the positive association between the expression level of miR-2682-5p and intraocular pressure in glaucoma patients remains inconclusive. Our findings of down-regulated miR-2682-5p in atropine-treated scleral fibroblasts suggested its potential role in ocular anti-remodeling as elevated IOP levels could cause ischemic effects and mechanical stress. Whether the regulation of miR-2682-5p in sclera is mediated by ocular growth and remodeling microenvironment merits further clarification.

The dose of 100μM of atropine was chosen with reference to most published in vitro studies 22,24,25. As shown in our NGS data, 100μM of atropine did not cause a dramatic change of gene expression in human scleral fibroblasts. Atropine eye drops have long been used for myopia control in children and adolescents 42. Recently, atropine eye drops at a lower-concentration of 0.01% has been found to be effective in preventing myopia progression as well. Subjects that received low concentration atropine had smaller accommodation amplitude reductions, and smaller pupil dilatations which could minimize the photophobia effects 17. From a prevention perspective, atropine at a dose of 100
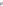
M, equivalent to 0.003%, could potentially induce alterations in the scleral remodeling process, fortunately, with slight changes in gene expression.

## Conclusion

In summary, our exploratory study indicated differentially expressed genes were enriched in anti-remodeling effects of scleral fibroblasts, including cell differentiation and structural changes. As atropine for myopia control is typically instilled before bedtime, functionally enriched pathways in inhibiting melatonin-degradation during the night may be partly responsible for reducing scleral remodeling. A focus on the miR-2682-5p-*KNCJ5* and miR-2682-5p-*PRLR* interactions presented a scientific basis to evaluate the participation of low-dose atropine treatment in scleral fibroblasts. The current findings may provide further insight into the benefits of atropine in preventing excessive ocular growth.

## Figures and Tables

**Table 1:** Genes selected between putative targets of microRNA and differentially expressed genes from NGS database

Up-regulated miRNA	Target down-regulated mRNA	Gene name	Fold change (Log_2_FC)
hsa-miR-193b-5p	*PLXDC1*	plexin domain containing 1	-1.38
hsa-miR-2114-5p	*SEMA6A*	semaphorin 6A	-1.16
hsa-miR-3074-5p	*CSRNP3*	cysteine and serine rich nuclear protein 3	-1.17
hsa-miR-3127-5p	*SOX6*	SRY-box 6	-2.27
hsa-miR-3158-3p	*GATAD2B*	GATA zinc finger domain containing 2B	-1.39
hsa-miR-3934-5p	*ZNF546*	zinc finger protein 546	-3.64
Down-regulated miRNA	Target up-regulated mRNA	Gene name	Fold change (Log_2_FC)
hsa-miR-1291	*FOXP3*	forkhead box P3	1.56
	*RNF43*	ring finger protein 43	1.82
	*CDT1*	chromatin licensing and DNA replication factor 1	1.57
	*MAP3K9*	mitogen-activated protein kinase kinase kinase 9	1.71
hsa-miR-2682-5p	*VPS53*	VPS53, GARP complex subunit	1.70
	*VIL1*	villin 1	1.47
	*PRLR*	prolactin receptor	1.29
	*PDE4B*	phosphodiesterase 4B	1.30
	*KCNJ5*	potassium voltage-gated channel subfamily J member 5	1.92
	*MAP3K9*	mitogen-activated protein kinase kinase kinase 9	1.71

**Table 2:** Gene Expression Omnibus verification of dysregulated genes in scleral fibroblasts

GEO Accession Number	GSE71743		GSE18811	
Specimen	Human (infant)		Human	
Tissue	Scleral fibroblast		Retinal pigmented epithelial cell	
	Posterior/anterior		Fetal/adult	
Genes	Up/Downregulation	*P*-value	Up/Downregulation	*P*-value
**Upregulated genes**				
*FOXP3*	DOWN	0.855	DOWN	0.069
*RNF43*	UP	0.190	DOWN	0.571
*VPS53*	DOWN	0.356	DOWN	0.056
***VIL1***	UP	0.189	**DOWN**	**0.003**
*CDT1*	UP	0.127	UP	0.208
***PRLR***	**DOWN**	**0.021**	**DOWN**	**0.008**
*PDE4B*	UP	0.029	DOWN	0.481
***KCNJ5***	DOWN	0.977	**DOWN**	**0.029**
*MAP3K9*	DOWN	0.064	UP	0.465
**Downregulated genes**				
*ZNF546*	UP	0.091	UP	0.279
*CSRNP3*	UP	0.133	DOWN	0.05
***SEMA6A***	**UP**	**0.013**	UP	0.211
*SOX6*	UP	0.119	UP	0.134
*GATAD2B*	UP	0.924	UP	0.118
*PLXDC1*	UP	0.554	DOWN	0.001

The genes and their directions of expression marked in **bold** were those that were expressed in opposing directions in both datasets, and the expression changes were significant in at least one.

**Table 3:** Potential miRNA regulations of identified genes in atropine treated scleral fibroblasts

Up-Regulated miRNA	Fold-Change	Predicted Target Down-regulated mRNA	miRmap Score	TargetScan Score	DIANA-microT
hsa-miR-2114-5p	2.14	SEMA6A	99.08	-0.04	-
Down-Regulated miRNA	Fold-Change	Predicted Target Up-regulated mRNA	miRmap Score	TargetScan Score	microT-CDS
hsa-miR-2682-5p	-2.32	PRLR	99.87	-0.28	0.908
		KCNJ5	98.93	-0.1	0.761

- indicated no miRNA-mRNA interaction predicted in database.
